# Clinical Empathy and Narrative Competence: The Relevance of Reading Talmudic Legends as Literary Fiction

**DOI:** 10.5041/RMMJ.10198

**Published:** 2015-04-29

**Authors:** John H. Davidson

**Affiliations:** Assistant Professor of Medicine, Mayo Medical School; Consultant, Division of Executive and International Medicine, Mayo Clinic, Rochester, Minnesota, USA

**Keywords:** Empathy, literature, narrative medicine

## Abstract

The “curative potential” in almost any clinical setting depends on a caregiver establishing and maintaining an empathic connection with patients so as to achieve “narrative competence” in discerning and acting in accord with their preferences and best interests. The “narrative medicine” model of shared “close reading of literature and reflective writing” among clinicians as a means of fostering a capacity for clinical empathy has gained validation with recent empirical studies demonstrating the enhancement of theory of mind (ToM), broadly conceived as empathy, in readers of literary fiction. Talmudic legends, like that of Rabbi Judah’s death, are under-appreciated, relevant sources of literary fiction for these efforts. The limitations of narrative medicine are readily counterbalanced by simultaneously practiced attention to traditional bioethical principles, including—especially—beneficence, non-maleficence, and autonomy.

On the day when Rabbi [Judah] died, the rabbis had decreed a public fast and offered prayers for heavenly mercy. They had furthermore announced that whoever said that Rabbi was dead would be stabbed with a sword. Rabbi’s handmaid ascended to the roof and prayed: “The immortals desire Rabbi to join them, and the mortals desire Rabbi to remain with them. May it be the will of God that the mortals may overpower the immortals.” When, however, she saw how often he resorted to the privy, painfully taking off his tefillin and putting them on again, she prayed: “May it be the will of God that the immortals may overpower the mortals.” As the rabbis continued their prayers for heavenly mercy, she took up a jar and threw it down from the roof to the ground. At that moment they ceased praying and the soul of Rabbi departed to its eternal rest.

(Babylonian Talmud, Tractate Ketubot 104a)

This well-known Talmudic legend is subtly evocative. On the one hand, it may be read as a hackneyed, sentimental tale condemning the strait-jacketed, ritualistic rabbis facing their leader’s imminent death in contrast to the more flexible, attentive handmaid who intentionally ends the prolonged dying process. On the other, it may be read as a complicated story with gaps which invite speculation as to the motivations of the rabbis and the handmaid. To read it in the latter fashion is to allow an evocation of a more nuanced view of the text which has relevance for a caregiver of any century past or present who faces the challenges of acting with clinical empathy and what has been termed narrative competence.

## CLINICAL EMPATHY

A recent consideration of clinical empathy is found in Jamison’s essay collection, *The Empathy Exams*, where she writes as both a medical actor who helps students learn interviewing techniques and as a patient herself.^1^ She begins:

Empathy comes from the Greek *empatheia*—*em* (into) and *pathos* (feeling)—a penetration, a kind of travel. It suggests you enter another person’s pain as you’d enter another country, through immigration and customs, border crossing by way of query …^1^

Jamison considers empathy to be an intentional state of insight for any clinician with regard to a patient, and more than “Checklist item 31” on the form which she fills out after each student interview. She details the consequence of an absence of empathy when recalling a complicated procedure of her own with an impatient cardiologist. Her implication is that the physician was at least neglectful if not lazy in the work of empathizing. She concludes:

Empathy isn’t just something that happens to us—a meteor shower of synapses firing across the brain—it’s also a choice we make: to pay attention, to extend ourselves … The act of choosing … means … “I will listen to his sadness, even when I’m deep in my own.”^1^

Not to listen invites bad medical outcomes.^2^,^3^ Groopman and Ofri have documented that medical mistakes result mainly from the interference of emotional factors not addressed during dialogues between clinicians and patients—a failure of empathic connection.^4^,^5^ In their view, this is a greater determinant of errors than knowledge deficits or technological incompetence per se.

Groopman’s and Ofri’s claims are consistent with observations of Ornstein, who posits that the “curative potential” in the doctor–patient relationship is dependent on the physician’s “empathic observation, empathic listening, and introspective self-awareness.”^6^ It is in clinicians putting “ourselves into the shoes of another” and viewing “his world … from his own vantage point,” while remaining attentive to our own.

Ornstein affirms Balint’s insight “that by far the most frequently used drug in general practice [is] the doctor himself.”^7^ In what dosage, form, and frequency is the doctor to prescribe a “professional self” without eliciting unwanted side effects?^6^ There is no formulaic pharmacologic text or algorithm for the dispensing of empathy. But when it is lacking, undesired outcomes may result. Consider the following:

During mid-morning rounds, our pulmonary consulting team approached the nurses’ station. An alarm sounded. We had been called a few hours earlier to review a patient’s status. He was becoming increasingly breathless and virtually speechless. Now he had arrested.

We had been told that he was an elderly grandfather suffering with a metastatic cancer which had left him in constant pain, frequently delirious, incontinent, and too weak to eat, drink, toilet, or walk without assistance. He was jaundiced and cachectic with a distended abdomen that made every breath a hungry gasp. He was a heap of skin, bones, and misery. He was without a code status because his family members could not agree, even though he had definite ideas.

The overhead intercom blared “Code Blue! Code Blue!” The crash cart drawers were opened. Cardiac compressions began. Sleep-deprived intensive care unit (ICU) residents burst onto the scene. “What’s the code status?” their leader barked. “There is none!” exclaimed the patient’s nurse. The room, already filled with the stench of stool, urine, and hospital disinfectant, was now suffused by uncertainty. Sweaty doctors and nurses hovered over the patient’s bed as one resident pushed on his chest and another squeezed the airbag.

“If there’s no code status, then we do everything,” the ICU resident announced. Shocks, drugs, bagging, and the rib-cracking compressions. Within moments, the patient’s attending physician arrived. She surveyed the scene and shouted, “Stop it! Stop all of this! Enough! He doesn’t want all of this!” The ICU resident countered, “But there’s no code status!” The attending physician responded, “The code status is that he is dying and suffering. We’re making it worse.” They stood face-to-face. He over six feet. She barely over five. “I take all responsibility,” she said. “Okay, then. Stop it,” he directed the team. The elderly grandfather died within minutes.

The attending physician had “crossed the border” to be with her patient more than once in the preceding days. She did so as one recognized for her astute knowledge of the principled, oft-cited tetrad of biomedical ethics: beneficence, non-maleficence, autonomy, and justice.^8^ But there was more. She understood his reluctance to undergo resuscitation and his hesitancy in addressing family members who disagreed. She had accepted a role as his advocate when the time came. He and she had inhabited their own borderland of empathy while making this and other clinical decisions. She had embraced the approach of Ornstein in seeking to practice “empathic observation, empathic listening, and introspective self-awareness.”^6^ The consequence was her being able to act with confidence in stopping the “Code Blue,” and avoiding a “medical error.” She did so, knowing that she would be required to explain the events and her patient’s request to his family. As a physician, what made her different?

## NARRATIVE COMPETENCE

It was her achievement of “narrative competence.” Charon succinctly defines this competence as “the ability to acknowledge, absorb, interpret, and act on the stories and plights of others.”^9^ She suggests that this skill is the essence of “humane and effective medical practice.” But how can it be taught, learned, and sustained as a professional value?

Over the past three decades, Charon and others have answered this question by creating a “narrative medicine” model within the milieu of routine clinical practice.^9^–^15^ It consists of shared “close reading of literature and reflective writing” among clinicians, as a means of catalyzing and sustaining their empathic connections with patients, colleagues, and other third parties.^9^ The programmatic structure of these efforts varies, but “concrete clinical sequelae” are held in common as interviewing “routines are turned on their heads,” with more open-ended statements like, “Tell me what you think I should know about your situation,” and notes beginning to take on new appearances with a “higher word-to-number ratio, longer stretches of ordinary language, affectively dense tone, and even the occasional use of the word ‘I’.”^14^

The narrative model has gained intriguing empirical validation as well from social science research examining the effects on volunteers of reading literary fiction. Kidd and Castano have demonstrated in sophisticated experiments that reading literary fiction, like that of National Book Award finalists and PEN/O. Henry Award winners, leads to better performance on tests for affective Theory of Mind (ToM) and cognitive ToM than does reading non-fiction, popular fiction, or nothing at all.^16^ The term ToM is here defined as “understanding others’ mental states” in the context of “the complex social relationships that characterize human societies,” broadly conceived as empathy, the essential catalyst for narrative competence.

Kidd and Castano have concluded that the enhancement of ToM, or empathy, which follows reading literary fiction, occurs because the text “forces us to engage in mind-reading and character construction,” “to enter a vibrant discourse with the author and her characters,” “to fill in gaps” in the storylines, and to hold “multiple perspectives” of characters and events simultaneously. They argue that this effort engages in the reader “psychological processes” which “as in real life” are “needed to gain access” to “complicated individuals whose inner lives are rarely easily discerned.”^16^ They cite the works of literary critic Roland Barthes and psychologist Jerome Bruner.^17^,^18^ An analogy of readers and texts to clinicians and patients is obvious. The enhanced scores after reading literary fiction and the derivative skill of “mind-reading” which they imply provide support for the model and methods of narrative medicine in fostering clinical narrative competence during patient care.

## LIMITATIONS OF NARRATIVE MEDICINE

While the model and methods of narrative medicine constitute a “uniquely effective approach to the challenges and questions of bioethics” and clinical practice, it is not “a perfect antidote” for the shortcomings of the oft-cited principalist tetrad of the former, or the sometimes overused checklist protocols of the latter.^8^,^19^,^20^ In the view of Cutter, the narrative approach should be seen as a “partner” to these alternatives and as producing “surprising new perspectives.”^20^ As the narrativist works “to capture the stories patients and families tell about the way they arrived at a particular predicament as well as the how of their moral decision-making at earlier important moments,” there is a clear focus first on “how we got here” before shifting to the “what to do” mode which is often the more primary concern of the bioethicist (and the checklist recorder).^21^ Nonetheless, even narrativists like Mitchell do not consider their efforts “sufficient for a stand-alone method” and admit “qualms” about taking this route exclusively without acknowledging potential “falling rocks” and “curve(s) ahead.”^22^

Specifically, Mitchell emphasizes that “not everyone is naturally narrative” in their outlook and some situations are better dealt with by other means. Secondly, she acknowledges that “stories (may) leave out and may conceal as much as they reveal” while “bleaching barely stated facts … of their authentic, chaotic, unreconstructed humanness” in the pursuit of a literary aesthetic. Thirdly, there can be “potentially pressuring aspects of allegedly therapeutic storytelling” in which patients and families are compelled “into telling us their stories when they might really rather not.” Fourthly, stories include not only reported phenomena from specific cases, but also “import moral assumptions and convey norms” as storytellers and interpreting clinicians seek to “persuade” others to “share their evaluation of the moral (and clinical) situation.” (This tendency may be seen in the interpreting of the Rabbi Judah story and will be considered below.) Lastly, Mitchell points out that there is an imperative sometimes unacknowledged by clinicians eliciting patient stories to be prepared for and not surprised by the complexity of emotional pain and cognitive dissonance which may arise. The vulnerable and expressive patient may be harmed by an inattentive response; by an unwelcomed false retelling of their story which distorts, diminishes, or oversimplifies; or even worse, by avoidance or desertion as if the clinician plays, “hot potato … to use a metaphor of children’s games,” by passing the patient on prematurely to another caregiver.^22^ In sum, Mitchell is observing that not everyone is a storyteller; not all stories are true; some stories are coerced; some stories are overly moralistic; and, telling stories can sometimes awaken considerable distress.

By recognizing these potential “falling rocks” and “curve(s) ahead” as limitations of narrative medicine, Mitchell has not primarily diminished the offerings of this method but has rather reinforced the ideal of partnering it with others, as Cutter has suggested.^20^,^22^ Clearly, while maintaining the more common, principled focus on beneficence, nonmaleficence, and patient autonomy, at the same time as pursuing narrative competence as described, clinicians will be less likely to coerce, to fabricate, to mislead, to compromise patient self-determination, or to increase the patient’s vulnerability. Just as the most compelling narrative invites a counter-narrative, so does the narrative method itself invite the counterbalance of a simultaneously practiced more traditional approach.

## RELEVANCE OF TALMUDIC LEGENDS

With these insights in mind, it is appropriate to continue with a consideration of the relevance of Talmudic legends, like the tale of Rabbi Judah’s death, as underutilized sources of literary fiction to be tapped along with the “selected literary works … by award-winning and canonical writers” cited in the studies above.^16^,^23^,^24^ These rabbinic texts impose an interpretive mandate and are clearly “writerly texts,” in the usage of Barthes, which beg the reader’s engagement as opposed to offering passive entertainment.^17^

Calderon observes that, in spite of what may seem to be an impenetrable style and structure, Talmudic legends comprise “texts that have the power to move people.”^25^ She calls for democratizing them with “barefoot reading” and demonstrates how the texts may be read within a plexus of individual lives—“births, rites of passage, arguments, revelations, heartbreaks, weddings, deaths.” She would surely concur with Biale that many of these stories are “a uniquely creative form of literature,” recognized to be “as great a contribution of the Jews to world literature as the Bible itself.”^26^ Like Calderon, he asserts that the tales “range over all human existence, from the most profound [issues], such as the meaning of suffering, to the most mundane, such as proper etiquette in the toilet.”^26^ They contend that Talmudic legends belong within the genre of literary fiction. Arguably, reading them may also enhance empathy and increase the likelihood of a clinician’s achievement of narrative competence.

For example, while contemporary clinician “barefoot readers,” like others, might first interpret the already-cited Rabbi Judah story as a simple-witted morality tale depicting the merciful courage of the handmaid and the uncaring, dogmatic rigidity of the rabbis, they might also come to a more variegated view on subsequent reading and discussion with others. They might wonder if the rabbis were praying out of genuine grief at the imminent loss of their teacher and not just from the jolting of their ordered religious worldview. Or, were they praying with reluctance out of a sense of obligation to or intimidation from him to make entreaties incessantly, “to do everything,” to run a rabbinic “Code Blue”? Why is he silent? Why does he not speak? He is Rabbi, after all. Is it the misery, embarrassment, or delirium of his illness alone, or is it also a morally significant cowardice bringing him to leave his death decisions to others? Is this only a glimpse of a male-dominated hierarchical rabbinic society so inwardly focused on its own ritualistic mantras that it ignores the outward stench attendant to disease and privies during morning prayers until a “lowly” woman, whose hands have likely applied salves to and wiped excrement from Rabbi’s body, climbs to the roof and screams, metaphorically, “Enough!” with the shatter of a hurled earthen jar? Or, may she also be a conflicted caregiver of an increasingly demanding elder, whose moans and cleanings she wishes to escape, even to the point of near-euthanasia, by putting an end to the interminable prayers with the crash of a jar? Was her ascension to the roof, like a latter-day women’s synagogue balcony, above the fray below, nearer the heavens above, not only for proximity to the celestial realm, but also to address her own conflicting impulses? Did she carry the jar up with her as the act was on her mind from the start?

By reading in this more discerning way, clinicians would be imagining the inner lives of these characters like patients. They would be reading the minds of the rabbis, Rabbi Judah, and the handmaid in the way of a physician striving for empathic connection so as to be narratively competent. They likely would be entering the story giving consideration to literary elements, like voice, character, plot, and denouement, and coming to realize that their textual explorations were analogous to embracing Ornstein’s and their own clinical goals of intentional “empathic observation, empathic listening, and introspective self-awareness.”^6^ Thus, by including the reading of Talmudic legends in routine clinical practice, “barefoot readers” might experience a reciprocal process by which their literary and caregiving efforts would enhance each other.

This potential reciprocity is demonstrated with a revisiting of the case of the elderly grandfather dying from metastatic cancer. There the possible benefits of a clinician’s bringing the acquired habits and insights of narrative medicine to the bedside are evident in the questions which might be raised. Like the Talmudic tale, the hospital drama was one of gaps and ambiguity. The staff and ICU residents surely felt more than a simple reluctance to step outside the norms of usual protocol. They, like the rabbis of the story, likely held conscious and unconscious, conflicted views as to the right course. Were they feeling faithful to their calling to preserve life and health? Or, were they causing harm by intervening? Had they not seen others “miraculously” survive such calamities? Why not now? What did the grandfather really want? Why, like Rabbi Judah, had he been effectively silent in not claiming an official code status and thereby burdening his caregivers with this biting limbo? Was this fair of him? Was it an acceptable professional burden to place upon the caregivers? And, like Rabbi Judah’s handmaid, was the attending physician only assuming a role of advocacy based in empathic connection and demonstrating narrative competence, or was she also acting out of ambivalence and fatigue, emotional and physical, in what had been a prolonged experience of end-of-life care? Were her motives also conflicted and meriting the consideration of others along with her own introspection?

This concatenation of relevant questions arising from the sparks and gaps of a real-life clinical event is analogous to the queries issuing forth from the Talmudic story. In both cases, the essential hermeneutic effort is one which strives for empathic connection and narrative competence—whether with patients, families, and caregivers in a “non-fiction world,” or with imagined personalities of a fictional Talmudic legend. This reciprocity allows the doing of one to enhance the capacity for the doing of the other. Therein lies the relevance of reading Talmudic legends as literary fiction while simultaneously striving for the clinical empathy and narrative competence at the bedside or in the office which constitute the “curative potential” in the doctor–patient relationship and the essence of “humane and effective medical practice.”^6^,^9^

## CONCLUSION

In conclusion, a physician’s “empathic observation, empathic listening, and introspective self-awareness” comprise the “curative potential” in almost any clinical setting.^6^ “Narrative competence,” which is “the ability to acknowledge, absorb, interpret, and act on the stories and plights of others,” follows from this beginning.^9^ Even without a formulaic textbook or algorithm, these skills are teachable, learnable, and sustainable, with the methods of a “narrative medicine” model consisting of shared “close reading of literature and reflective writing.”^9^–^15^ This approach has gained credibility with recent empirical studies demonstrating the enhancement of ToM, broadly conceived as empathy, in readers of literary fiction which, arguably, may include Talmudic legends, like that of Rabbi Judah’s death above.^16^,^23^–^27^ While the limitations of narrative medicine are acknowledged, they are seen as counterbalanced by partnering narrative approaches with simultaneously practiced attention to traditional bioethical principles, including— especially—beneficence, non-maleficence, and autonomy.^8^,^20^,^22^

## Abbreviations:

ICU: intensive care unit;
ToM: theory of mind.
